# Contingency Nursing Management in Designated Hospitals During COVID-19 Outbreak

**DOI:** 10.5334/aogh.2918

**Published:** 2020-06-29

**Authors:** XiaoYing Wu, ShaoYan Zheng, JiaJia Huang, ZeLi Zheng, ManQing Xu, YuHua Zhou

**Affiliations:** 1 ShanTou Central Hospital, CN

## Abstract

**Background:**

In December 2019, early cases of COVID-19 were identified in Wuhan, China. By late January 2020, it was evident that COVID-19 was rapidly spreading and represented a national health emergency. In order to contain the spread of COVID-19, China adopted a centralized treatment plan by appointing designated hospitals in each region. Shantou Central Hospital is a Grade A Class A general hospital in Guangdong Province. It was appointed as a provincial COVID-19 designated treatment hospital on January 21, 2020, to provide all COVID-19-related treatments for the city of Shantou. The nursing department at Shantou Central Hospital is fully responsible for hospital nursing administration, nursing human resource management, nursing quality management, and all nursing tasks related to hospital medical care, nursing, teaching, scientific research, preventive healthcare, and so on.

**Objective:**

To summarize the role of nursing management in transforming a general hospital into a designated hospital for treatment of COVID-19 patients.

**Methods:**

We undertook a series of nursing management measures in the strategic phase and the implementation phase.

**Findings:**

Through a series of nursing management measures, all COVID-19 patients admitted to our hospital were cured and discharged. All non-COVID-19 patients and staff hospitalized during the same period were not infected with the virus. During this period, our hospital completed 7,466 operations. Hence, our nursing management measures were effective.

**Conclusions:**

Our efficient nursing management system, first of all, effectively mobilized all available manpower; secondly, up-skilled and trained personnel within a very short period of time; thirdly, provided reliable logistical support for front-line protection equipments; and finally, motivated nurses during this very difficult time to make a significant positive contribution to the fight against COVID-19 pandemic.

## Introduction

The main transmission routes of Corona Virus Disease-2019 (COVID-19) are respiratory tract and close contact. The elderly and people with co-morbidities are more susceptible to the virus as compared to the younger and healthy people and can develop severe disease [1]. COVID-19 spread rapidly in China. Based on the experience from SARS virus in 2003, after human-to-human transmission was confirmed [2], COVID-19 was treated as a national health emergency in China. As an acute respiratory infectious disease, COVID-19 has been included in category B, infectious diseases, as stipulated by the laws of the People’s Republic of China on the Prevention and Control of Infectious Diseases, and is managed as a category A infectious disease [1]. Strengthening the nursing management, protection, and isolation measures and improving nurses’ ability to respond to the pandemic are the tasks of hospital nursing management, especially for a COVID-19 designated treatment hospital such as ours, which also has to fulfill the existing general hospital functions.

## Background

Shantou is a city in southeastern China, which covers an area of 2198.7 square kilometers and has a total population of more than five million people [3,4]. Shantou Central Hospital is located in the center of Shantou. It is one of the 30 Grade A hospitals in Guangdong Province. Currently, it has more than 2,650 employees, including 1,506 nurses. The total constructed area is 150,000 square meters, and the number of open beds is 2,040. It is divided into four areas: medical area, surgical area, comprehensive ward, and outpatient and emergency area, with a total of 63 nursing units. Each nursing unit appoints 1–3 head nurses based on the number of patients admitted.

According to the requirements of the National Health Council of the People’s Republic of China, COVID-19 patients are treated intensively and quarantined together in the same hospital. Shantou Central Hospital was designated as the provincial COVID-19 rescue hospital in Shantou. This appointment is based on the fact that Shantou Central Hospital is not only one of the best-equipped with advanced facilities in the region but also has the most experienced doctors and nurses.

## Strategy

To transform a general hospital into a designated COVID-19 treatment hospital and maintain the high standard of care for each patient. The nursing department planned the following contingency management strategic objectives.

- Set up designated COVID-19 wards.
- Establish a technical support team.
- Ensure the hospital has ready and available reserve nurses.
- Prepare training plan to meet all requirements.

### Setting up wards for patients with COVID-19

The nursing department collaborated with other functional departments to set up wards exclusively for COVID-19 patients. The hospital leaders designed the layout of the wards for COVID-19 patients such that they were isolated from other parts of the hospital. The necessary logistics in/out or within these wards have strict guidelines to minimize any possible cross-infections. The hospital has designated the fifth floor of the original infection ward for patients with severe COVID-19. The wards on the fourth floor are dedicated to the treatment of mild COVID-19 patients. The second and third floors of the original infection department are used as the COVID-19 medical observation ward. The entire floor of the cardiac intensive care unit (CCU) was emptied and disinfected to prepare for the admission of critically ill patients. Meanwhile, the original tumor ward building was quickly renovated as the second ward for COVID-19 patients. The chief nursing officers conducted on-site inspections to approve the layout. Particular emphasis was laid on the organization and separation of the contaminated areas, semi-contaminated areas, and clean passages to ensure clear instructions are followed by personnel in and out of these wards; ensure all the items in each ward are exclusive for that ward till their safe disposal; and that there are special toilets and bathrooms in each ward.

### Establish a technical support team

The nursing technical support team for COVID-19 is comprised of the head nurses of the infection department, emergency department, intensive care unit (ICU), CCU, and respiratory department, led by the chief nursing officer. The nursing technical support team is responsible for providing consultation, guidance, and nursing technical support for COVID-19. The nurse on duty every day in the isolation ward updated the patient’s condition on the WeChat group of the COVID-19 nursing technical support team, based on which the team members made timely nursing recommendations. The team members regularly summarized and reported the admission and treatment of the whole ward, adjusted the focus of the nursing work in a timely manner, and formulated new nursing work content and procedures in a very efficient manner according to new developments. The team leader adjusted the staffs in a timely manner to ensure optimal allocation of human resources and also ensure that the nurses were not overwhelmed by unexpected events that may cause chaos in the hospital. The team leader participated in regular meetings of the COVID-19 treatment and protection leading group in the hospital every day. This helped the team leader to understand the dynamics of the treatment and protection of COVID-19 patients in the hospital, to propose other functional departments that could help solve difficult problems that required immediate attention, and to help the nursing management ensure smooth progress of nursing work during the pandemic. The technical support team facilitated collaboration not only within designated COVID-19 wards but also across all functions of the hospital. In the time of crisis, all available resources were focused into frontline to deliver the highest standard of care to all patients.

### Ensure the hospital has ready and available reserve nurses

Managing uncertainty is the biggest challenge in a time of crisis. Human resource planning was particularly challenging for the nursing department, which had to ensure sufficient manpower in case of a sudden increase in the number of patients, while maintaining the same level of service to all other functional departments. To ensure normal nursing service in all other wards in the hospital, the nursing department requested each department to nominate 1–2 nurses as a COVID-19 ward backup nursing team. Although this was a voluntary process, 82 nurses had registered, of which 24 with work experience in the infection department, emergency department, or ICU were selected to form the first manpower echelon of backup nursing and were trained in isolation and protection guidelines. After they successfully completed the assessment, they were stationed to support the infection isolation ward and the emergency fever pre-diagnosis area. The remaining nurses served as the second backup nursing human echelon. They were on call after training and were required to make themselves available to support the infection isolation ward or emergency department at any time.

### Organized training for all employees, on-site training, and online training protection and isolation knowledge

The combination of on-site training and online training was used to maximize the hospital infection prevention, and control awareness and capabilities of medical staff. On-site training was a priority for the infectious disease control department. Critical training, including COVID-19 hospital infection prevention and control, hospital air purification management specifications, medical institution disinfection technical specifications, and personal protection requirements for disinfection and isolation, was provided for all nurses working in the infection control department before encountering any patient with COVID-19. The nursing department also organized relevant departments to conduct emergency drills, including patient admission procedures, negative pressure flat car transporting patients, the process of patient consultation, and treatment procedures. The online training included uploading the coursework and lecture video of the COVID-19 hospital prevention and control on the hospital’s WeChat group and mobile phone application, and all nurses were required to complete the training as soon as possible. These training sessions were comprehensive and detailed, including putting on and taking off gowns and washing hands. Instructive signage was displayed on every dressing room and hand washing area throughout the hospital as a reminder, to ensure best hygiene practices were followed by all the people who worked and visited the hospital.

## Implementation

### Ensure normal operation of medical care in the hospital

To ensure normal operation of all other functional departments in the hospital, the hospital revised 32 items on its procedures including the work guidelines and emergency plans for the protection and isolation of COVID-19 infections, ward disinfection and isolation systems, and terminal disinfection process guidelines. The nursing department was tasked with implementing the revised family and patient admission system. All personnel entering the hospital are required to wear masks and undergo body temperature tests, and every ward’s doors are affixed with “Advice on Visiting and Visiting Our Hospital Ward”. There is only one gate to allow personnel to access each ward during the pandemic, and the number of visiting staff and visiting time is strictly controlled. It’s not recommended to visit patients in the hospital. Each hospitalized patient is limited to one fixed escort whose COVID-19 nucleic acid test displayed negative results. Under special circumstances, the patient must obtain the permission of the ward manager. Before entering the ward, all visitors must register the following information: name, age, whether they have visited Wuhan and neighboring areas within the past 14 days, and whether they had contact with COVID-19 infection (positive nucleic acid test) within 14 days, fever, body temperature ≥37.3°C, cough, or other respiratory symptoms [1]. We also recommend that non-emergency patients try to avoid hospitalization during the COVID-19 pandemic. These measures are strictly enforced throughout the hospital to minimize any possibility of cross-infection and reduce disruption to other functional departments.

### Manage nursing manpower in isolation wards

Within the isolation wards for COVID-19, the nursing department had reallocated all manpower. The mild patient areas were managed by the original head nurse of the infection department and the original intensive care team relocated to the area of critical patients. The head nurse formulated the job responsibilities of the COVID-19 serious patient care team and the matters needing the attention of the COVID-19 serious medical care team (Table 1). The job responsibilities were adjusted based on changes in the nursing work process. Each nurse was restricted to work no more than four hours a day to prevent physical overwork. Significant developments, such as a change in the number of patients, were reported to the head nurse and the most appropriate action was taken, for instance, to call up reserve nurses [5].

**Table 1:** Nursing duties of each shift.

Shift type	Operating hours	Nursing duties

A1	08:00–12:00	Check the operation of high-flow oxygen therapy machines and ventilators, check indwelling needles and infusion therapy
A2	11:00–15:00	Check the operation of high-flow oxygen therapy machines and ventilators, assist the patients in eating, taking medicine, scrub baths, perineal scrubs, and changing contaminated clothing
dcB1	14:00–18:00	Check the operation of high-flow oxygen therapy machines and ventilators, scrub bath, perineal scrub, change contaminated clothing, and treatment
B2	17:00–21:00	Check the operation of the high-flow oxygen therapy machines, assist the patients in eating, taking medicine, and treatment
C1	20:00–24:00	Check the operation of the high-flow oxygen therapy machines and ventilators, wipe the table with 75% ethanol wipes, dispose the ward garbage, check the operation of the disinfection machine in each area, and arrange for the patients to rest
C2	23:00–03:00	Check the high-flow oxygen therapy machines and ventilators, check whether the bedside items are complete (large and small urinals), hot water cups, prevent the patients from falling down, check the ward items, and check for leaks
D1	02:00–06:00	Check the operation of the high-flow oxygen therapy machines and ventilators, check whether the bedside items are complete (large and small urinals), hot water cups, prevent the patients from falling down, check the ward items, and check for leaks
D2	05:00–09:00	Check the operation of high-flow oxygen therapy machines and ventilators, draw blood from patients, morning care (assist patients with washing their faces, brushing their teeth, etc.), assist in eating and taking medicine
A3	08:00–15:00	Inventory of medicines, prescriptions, doctor’s orders, helping the responsible nurse to complete the nursing records, ordering meals, cleaning goggles, and drying goggles for later
B3	14:00–21:00	Replenishing medicines, supplies, liquids in the dispensing room, supplement protective equipment, and assist the responsible nurse
C3	20:00–03:00	Write nursing records to assist responsible nurses
D3	02:00–09:00	Write nursing records, upload shift content on WeChat group, and assist responsible nurses

### Provide a resting place for all nurses who had direct contact with COVID-19 patients

The nurses who entered the isolation wards to nurse COVID-19 patients were relatively fixed. They were provided with onsite accommodation, food, and all essentials. A centralized living resident health protection management method was also created, so that all nurses who were in direct contact with the COVID-19 patients would not have contact with outside personnel, in order to avoid cross-infection caused by medical staff.

### Establish psychological counseling via WeChat group

The rapid spread of COVID-19 and the general susceptibility of the population causes psychological pressure on the frontline nurses working in the ward. The nursing department conducted psychological interventions for frontline nurses and their family members, set up a WeChat group for psychological counseling, and invited psychiatrists at our hospital to join the group, in order to raise mental health awareness and directly address mental health concerns of frontline nurses and their family by experts, free of charge. Often, positive stories and successful cases were shared on the WeChat group, which motivated everyone at the hospital [6].

### Equip isolation ward with isolation protection materials

Due to the rapid spread of the pandemic, there could be a shortage in the supply of protective equipment that was not used frequently in the past, especially personal medical protective equipment, such as gowns, protective glasses, N95 masks, and the like. The nursing department horizontally coordinated with the materials department and the head nurse of the isolation ward to make a usage plan by doing everything possible to equip the isolation ward with protective materials and equipment to ensure that the isolation ward has sufficient supplies [7], so that the frontline nurses can perform their nursing work with peace of mind.

## Results

Since January 21, 2020, 75 patients suspected to have COVID-19 infection were admitted to our hospital, and 12 cases were confirmed by testing. By March 7, 2020, all patients had been cured and discharged. During the treatment of COVID-19 patients in the hospital, we closely observed all inpatients in the hospital and conducted COVID-19 nucleic acid screening for all the inpatients in the same period. All staff involved in the treatment were required to stay in the centralized station provided by the hospital for 14 days after discharge of all COVID-19 patients. During this period, all patients and staffs showed no symptoms of COVID-19 and their viral nucleic acid tests were negative. During the COVID-19 outbreak, the other departments of the hospital were functioning normally. From January to April 2020, the hospital performed a total of 7,466 operations, a decrease of 13.98% over the same period last year. This was related to the hospital’s recommendation for non-emergency patients to postpone their treatment, due to reasonable nursing manpower arrangements; there was no report of a shortage of nursing staff in any department in the hospital.

## Conclusion

During this pandemic, we realized the unforeseen challenges to the nursing management department. The sound contingency management system could effectively mobilize all available manpower; up-skill and train personnel in a very short period of time; provide reliable logistical support for frontline protection equipment; motivate nurses at this very difficult time; and thereby make a significant positive contribution to the fight against COVID-19. Use of the internet to communicate our message to all nurses and conduct remote training was new for us, and it played a tremendous role in equipping all personnel with relevant knowledge for combating the disease. The rapid spread of the pandemic and the limited space at the hospital were major challenges for the nursing management at our non-communicable diseases’ specialist hospital. In the case of the small number of critically ill patients, the critical care team and rescue equipment are moved to the infection department for nursing work. Instead of transferring critically ill patients to the intensive care department, the hospital maximized the space for COVID-19 infection patients to centrally seek treatment and reduce the patient flow in the hospital [8]. It also reduced the consumption of hospital resources and costs.
